# Non-Coding RNA as Biomarkers for Type 2 Diabetes Development and Clinical Management

**DOI:** 10.3389/fendo.2021.630032

**Published:** 2021-09-17

**Authors:** Tiange Chi, Jiaran Lin, Mina Wang, Yihan Zhao, Zehuan Liao, Peng Wei

**Affiliations:** ^1^School of Traditional Chinese Medicine, Beijing University of Chinese Medicine, Beijing, China; ^2^First Clinical Medical College, Beijing University of Chinese Medicine, Beijing, China; ^3^Department of Nephrology and Endocrinology, Dongzhimen Hospital Affiliated to Beijing University of Chinese Medicine, Beijing, China; ^4^Graduate School, Beijing University of Chinese Medicine, Beijing, China; ^5^Department of Acupuncture and Moxibustion, Beijing Hospital of Traditional Chinese Medicine, Capital Medical University, Beijing Key Laboratory of Acupuncture Neuromodulation, Beijing, China; ^6^School of Biological Sciences, Nanyang Technological University, Singapore, Singapore; ^7^Department of Microbiology, Tumor and Cell Biology (MTC), Karolinska Institutet, Stockholm, Sweden

**Keywords:** ncRNAs, miRNAs, lncRNAs, circRNAs, diabetes, biomarker

## Abstract

Diabetes, a metabolic disease characterized by high blood glucose and other complications, has undefined causes and multiple risk factors, including inappropriate diet, unhealthy lifestyles, and genetic predisposition. The two most distinguished types of diabetes are type 1 and type 2 diabetes, resulting from the autoimmune impairment of insulin-generating pancreatic β cells and insulin insensitivity, respectively. Non-coding RNAs (ncRNAs), a cohort of RNAs with little transcriptional value, have been found to exert substantial importance in epigenetic and posttranscriptional modulation of gene expression such as messenger RNA (mRNA) silencing. This review mainly focuses on the pathology of type 2 diabetes (T2D) and ncRNAs as potential biomarkers in T2D development and clinical management. We consolidate the pathogenesis, diagnosis, and current treatments of T2D, and present the existing evidence on changes in multiple types of ncRNAs in response to various pathological changes and dysfunctions in different stages of T2D.

## 1 Introduction

Diabetes is a kind of metabolic disease characterized by a high level of blood glucose with multiple complications, including macrovascular complications (cardiovascular disease) and microvascular complications (e.g., diabetic kidney disease, diabetic retinopathy, and neuropathy), and higher risks of developing several types of cancer, which subsequently could result in decreased life quality and even death (1–8). Type 1 and type 2 diabetes are the two most common types of this disease. Type 1 diabetes (T1D) presents an absolute insulin deficiency resulting from the autoimmune impairment of insulin-generating β cells, while type 2 diabetes (T2D) displays a relative insulin deficiency due to metabolic dysfunction (9). According to the estimation of International Diabetes Federation, the prevalence of diabetes increases dramatically; by 2045, diabetes is projected to affect approximately 700 million people worldwide, up from the previous estimation of 463 million in 2019. Moreover, approximately one in every two persons living with diabetes is undiagnosed. In 2019, diabetes causes over 4 million deaths globally in the 20–79 years age range (10, 11). To be diagnosed as diabetic, one’s blood glucose should be equal to or above certain values. According to the classification and diagnosis of diabetes by the American Diabetes Association (12), the methods and criteria are as follows: fasting plasma glucose test [FPG ≥126 mg/dl (7.0 mm/L)] and fasting are defined as no caloric intake for the past 8 h at least; 2-h oral glucose tolerance test [OGTT ≥ 200 mg/dl (11.1 mmol/L)] and the test should be conducted strictly as described by WHO, using a glucose load containing the equivalent of 75 g anhydrous glucose dissolved in water; glycated hemoglobin test [A1C ≥ 6.5% (48 mmol/mol)]; or random plasma glucose ≥ 200 mg/dl (11.1 mmol/L), coupled with classic symptoms of hyperglycemia or hyperglycemic crisis. In the absence of definitive hyperglucose, at least two of the abnormal test results are required for the diagnosis of diabetes, either from one same sample or two separate ones.

Non-coding RNA (ncRNA) refers to a type of RNA that is not involved in producing proteins but plays a key role in cellular function and development of different diseases. More than 90% of human genome RNAs consist of ncRNAs, and they can be divided into several types based on their size (13). The small ncRNAs such as microRNAs (miRNAs) and longer ncRNAs including long non-coding RNAs (lncRNAs) have been discovered that their up- or downregulation may regulate endothelial function in the vasculature, which is associated with the occurrence of diabetes (**Table 1**) (50). Moreover, the development of islet autoimmunity and dysfunction of β cells may result from the deregulation of immune-cell-specific T1D loci-associated lncRNAs and islet-specific lncRNAs, which leads to T1D (51). In addition, lncRNAs are linked to poor glycemic control, insulin resistance, senescence, and proinflammation in patients with T2D (45). Thus, the specificity that ncRNAs exert significant functions in adjusting cellular pathways and the development of diseases affects their expression patterns, which is reflected in various body fluids, make them ideal as biomarkers for diabetes. This review aims to consolidate the pathogenesis, diagnosis, and treatments of T2D, and the role of ncRNAs as biomarkers in progress and management of T2D.

**Table 1:** Summary of different expressions of ncRNAs in various target cells of T2D and prediabetic patients.

	Differentially Expressed ncRNAs	Target Cells	Reference
**Enhanced↑**			
**T2D Patients**	miR-16	Pancreatic β cells	(14)
	CDR1	Pancreatic β cells	(15, 16)
	circRNA-HIPK3	Pancreatic β cells	(16)
	hsa_circ_0054633	Pancreatic β cells	(17)
	circANKRD36	Pancreatic β cells	(18)
**Prediabetes Patients**	miR-499-5p	Hepatic cells	(19, 20)
	hsa_circ_0054633	Pancreatic β cells	(17)
**Reduced↓**			
**T2D Patients**	miR-376	Pancreatic β cells	(21)
	miR-432	Pancreatic β cells	(21)
	miR-200	Pancreatic β cells	(22)
	miR-184	Pancreatic β cells	(23)
	miR-204	Pancreatic β cells	(24)
	miR-24, miR-26, miR-148, miR-182	Pancreatic β cells	(25)
	miR-9	Pancreatic β cells	(26)
	miR-130a, miR-130b, miR-152	Pancreatic β cells	(27)
	miR-187	Pancreatic β cells	(28)
	miR-7	Pancreatic β cells	(29)
	miR-708	Pancreatic β cells	(30)
	miR-34a, miR-146a	Pancreatic β cells	(31, 32)
	miR-182-5p, miR-33, miR-37	Pancreatic β cells	(33, 34)
	miR-802	Hepatic cells	(35, 36)
	miR-122-5p	Hepatic cells	(37)
	miR-106b	Skeletal muscle cells	(38, 39)
	microRNA let-7a, let-7d	Skeletal muscle cells	(40)
	miR-29	Skeletal muscle cells	(41)
	miR-192, miR-122, miR-27a-3p, miR-27b-3p	Adipocytes	(42)
	LncRNA H19	Pancreatic β cells	(43, 44)
	LncRNA MEG3	Pancreatic β cells	(45–47)
	LncRNA MALAT1	Pancreatic β cells	(48, 49)

## 2 Type 2 Diabetes

### 2.1 Current Pathogenesis

According to the American Diabetes Association (ADA) (12), diabetes can be classified into the following general categories: type 1 diabetes; type 2 diabetes; gestational diabetes mellitus; specific types of diabetes due to other causes, e.g., monogenic diabetes syndromes (such as neonatal diabetes and maturity-onset diabetes of the young) and diseases of the exocrine pancreas (such as cystic fibrosis and pancreatitis); and drug- or chemical-induced diabetes (such as with glucocorticoid use, in the treatment of HIV/AIDS, or after organ transplantation).

T2D is characterized by hyperglycemia, insulin resistance, and relatively impaired insulin secretion. Ever since the study of the impaired responsiveness to insulin of the diabetics opened by Berson and Yalow in the 1960s (52), insulin resistance has been proposed, investigated, demonstrated, and concluded as the initial defect in T2D. To compensate for the insufficient insulin functions due to the presence of insulin resistance, hyperinsulinemia occurs, consequent to increased β-cell insulin secretion. However, it is worth noting that patients with primary insulin resistance, characterized by marked hyperinsulinemia and genetically dysfunctional insulin receptor, namely, those with type A insulin resistance, Rabson–Mandenhall syndrome, or Leprechaunism, may have close to normal glucose tolerance, retain normal weight, and normotrygliceridemic, in spite of congenital significantly elevated plasma insulin concentrations (53). Therefore, it is the secondary insulin resistance that is being discussed here, which is remarkably associated with T2D. In a popular context of chronic energy surplus, usually caused by sedentary lifestyle, adipocyte dysfunction may arise as a result of fibro-inflammation process, when white adipose tissue fails to properly adapt and expand in response to positive energy balance, which is normally induced by insulin, liver, pancreas, and skeletal muscle, T2D occurs (54).

It is widely assumed that aside from the aged tendency of population, changes in diet and lifestyle are also responsible for the speedy boost in the global prevalence and incidence of type 2 diabetes in recent decades. These two factors also contribute largely to the ongoing global obesity epidemic, while obesity is tightly correlated with the incidence of T2D (55). Epidemiological studies have shown that the level of overall physical exercise is related to a decline in the relative risk of diabetes by roughly 30% (56). There are also some evidence that T2D may derive from infection. For example, *Chlamydia pneumoniae* may induce β-cell dysfunction in the case of systemic inflammation (57). Although lifestyle and overeating seem to be the activating factors, genetic factors also play an important role in the pathogenesis of T2D. GWAS published in 2007 identified six new diabetes susceptibility genes: SLC30A8, HHEX-IDE, CDKN2A/2B, IGF2BP2, CDKAL1, and FTO (58–62). The first GWAS (61) repeated the previously known correlation between TCF7L2 and type 2 diabetes, which has been found in Icelandic populations (63). TCF7L2 is the most replicated genetic variant related to T2D so far, with a relative risk of 1.4. Besides, epigenetic factors (such as DNA methylation) are particularly crucial as they may mediate the impact of environmental exposure to T2D (64).

## 3 Different Types of ncRNAs Used as Biomarkers of Diabetes

**Figure 1:**
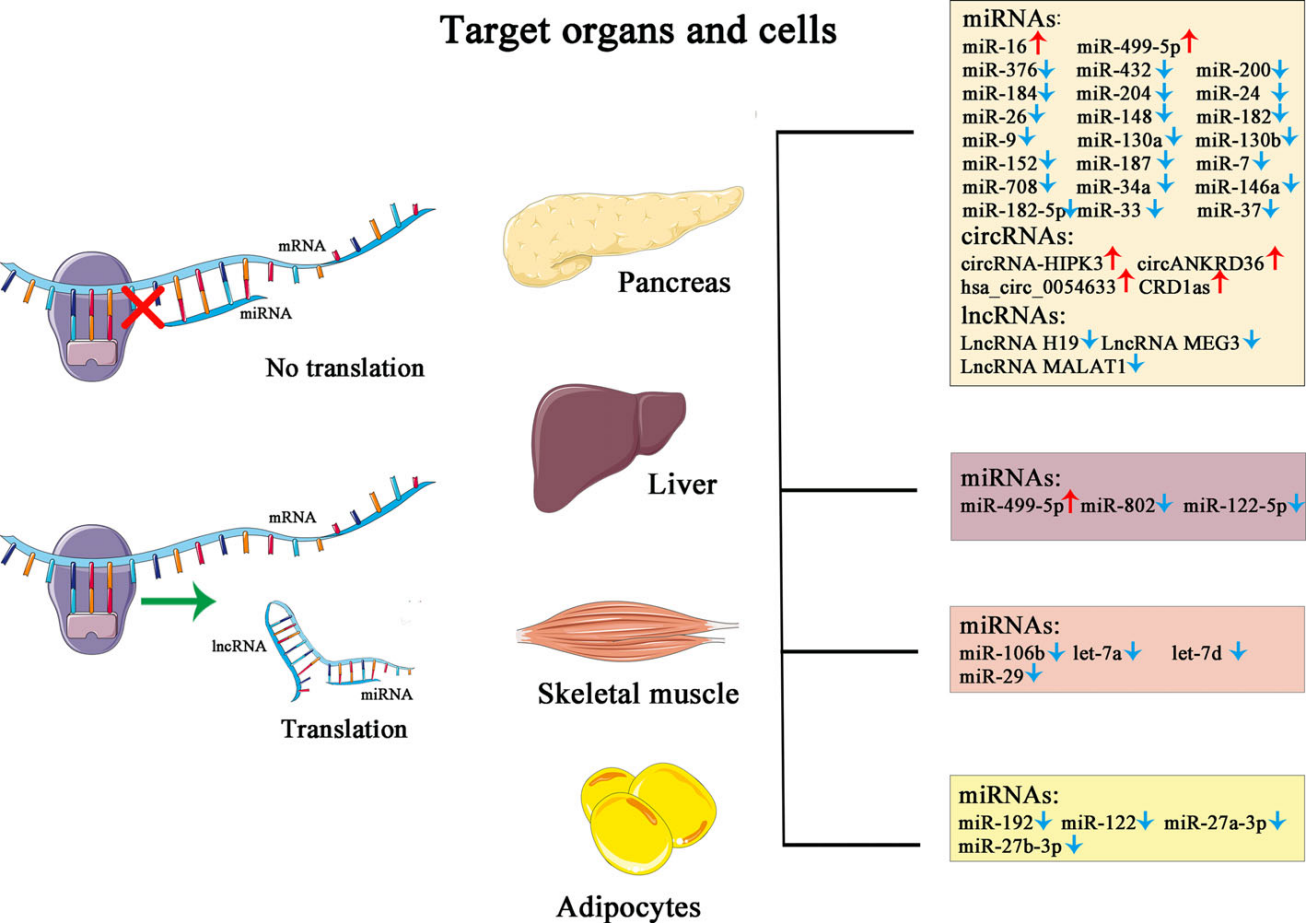
This figure illustrates the simplified mechanism of miRNA and lncRNA in post-transcriptional modulation of gene expression, the target organs and cells, and their corresponding changes in the pathological state of diabetes.

Non-coding RNA (ncRNA) is a functional RNA molecule that is not translated into a protein. The ncRNAs, making up more than 90% of human genome RNAs, were once labeled “molecular fossils” or “relics” due to their conserved nature and for being “useless” transcriptional products. However, countless significant functions have been unveiled during the past decades, including recruitment of epigenetic modifier proteins, control of mRNA decay and translation, and DNA sequestration of transcription factors, showcasing tremendous biological and medical potential with fast-gaining momentum (65). Categorized by size and length, members of ncRNA family include miRNAs, siRNAs, piRNAs, snoRNAs, snRNAs, exRNAs, scaRNAs, and the long ncRNAs, and the nonlinear circular RNAs. Furthermore, ncRNAs such as miRNAs, lncRNAs, and circRNAs have been proven to have tight links or direct participation in the pathogenesis, development, and prognosis of T2D. Due to their unique roles in modulating biological actions preceding the changes on glucose level and improved detectability and accuracy boosted by technological progression, ncRNAs are emerging as potent biomarkers in the diagnosis of the development and clinical management, utilized alone or complementary to the traditional yardsticks (**Figure 1**).

### 3.1 miRNA

miRNAs are a large group of small (15–22 nts), non-coding sequences with hairpin conformation, and are highly conserved among the species. Their major roles include direct posttranscriptional repression or cleavage of mRNA targets, resulting in the destabilization of the transcripts, functioning as a critical regulator of an overarching array of cellular processes such as cell development, proliferation, differentiation, apoptosis, and metabolism (66). miRNAs have underestimated potentials, owing to technological limitations. It is only until the discovery of two miRNAs, lin-4 and let-7, which occupies pivotal niches in the timing of development of *Caenorhabditis elegans*. There are over 2,000 miRNAs listed for *Homo sapiens*, and it is predicted that approximately 60% of protein-coding human genes possess miRNA target sites (67, 68).

#### 3.1.1 miRNA and Pancreatic β Cells

The orchestra between pancreatic β cells, insulin, and peripheral recipient cells including hepatic cells, skeletal muscle cells, and adipocytes is key to the occurrence of T2D. Therefore, apart from the conventional focus on the insulin insensitivity of recipient cells where blood glucose fails to enter or being stored as glycogen, attention should also be placed on the secretion and transportation of insulin as well. It has been proven that defective insulin secretion shares association with both reduced β-cell mass and impaired β-cell function, where miRNAs exert effect on (69).

The secretory functions of β cell relies on its physical existence, with emphasis on the proliferation and apoptosis thereof. In human T2D islets, the incidence of methylation of the DLK1-MEG3 cluster is high. This region contains cell-specific histone modifications and is home to over 50 miRNAs, among whom miR-376 and miR-432 has been exemplified to control islet amyloid polypeptide (IAPP) level, which is involved in β-cell apoptosis (21). It has also been found that the expression of miR-200 is highly induced in the islets of diabetic mice, whose beta-cell-specific overexpression suffices to induce beta cell apoptosis and lethal T2D, while its ablation can rescue β cells against apoptosis (22). The downregulation of miR-184 has been witnessed both *in vitro* and *in vivo* in obese diabetic mice, and a decreased expression in the islets of T2D patients, which corroborates the inhibitory effect of miRNA on beta-cell proliferation (23). Another phenomenon concerning beta-cell survival worth mentioning is the extracellular miR-16, which is delivered to β cell by exosome-like vesicles released by skeletal muscles in insulin-resistant conditions. Probably as a compensatory effect, the upregulated expression of said miRNA is in favor of insulin-secreting cell expansion, which increases islet size (14).

Intact as the cell structure may be, pancreatic β cell loses its existential significance once the secretory function proves useless. Before reaching target cells, insulin has to be synthesized and transported out of β cells. In diabetic mice models and cultured beta cells, miR-204 expression has been demonstrated to be induced, which blocks insulin production by directly targeting and downregulating MAFA, a known insulin transcription factor (24). A similar effect has been witnessed in line with the downregulation of a cohort of miRNAs, namely, miR-24, miR-26, miR-148, and miR-182, in isolated islets and cultured β cells where insulin content has been shown to be decreased due to reduced insulin promoter activity (25). MiR-9, among the first detected islet miRNAs, is one of the modulators of insulin granule exocytosis. Its increased expression represses the transcription factor ONECUT2 and increases the expression of granuphilin, which negatively regulates of insulin exocytosis (26). Intracellular energy production also plays a role in the secretion of insulin granules. miR-130a, miR-130b, and miR-152 are found to have overexpression in T2D patient islets, where they each reduced the level of the common target pyruvate dehydrogenase E1 alpha 1 subunit (PDHA1), thereby reducing intracellular ATP and insulin secretion (27). According to a global profiling of islet miRNAs in cohorts of individuals with and without T2D conducted by Locke et al., a dramatic increase in the expression of miR-187 in the islets obtained from T2D donors was noticed. Its exact mechanism on insulin secretion remains to be precisely defined; however, certain relations with homeodomain-interacting protein kinase 3 (HIPK3) were suspected, a protein kinase that is required for normal insulin secretion and a direct target of this miRNA (28).

miR-7 is abundantly expressed in pancreatic islet cells. Studies showed that miR-7 directly regulates insulin granule exocytosis by controlling late stages of insulin granule fusion with the plasma membrane and ternary SNARE complex activity, with no effect on cell proliferation and apoptosis (29). Transgenic mice overexpressing miR-7a in β cells developed diabetes due to impaired insulin secretion and β cell dedifferentiation, while in human, its pattern of expression oscillates responding to the extent of insulin resistance: it is reduced under moderate insulin resistance conditions, contributing to improved insulin secretion; however, it raises progressively under severe diabetic conditions and can reach levels even higher than in healthy individuals (29). The abovementioned phenomenon has established itself as a major obstacle in applying the expression of miR-7 as an independent or complementary biomarker in determining the diagnosis of T2D. In addition to that, challenges for the clinical application of miRNAs as biomarkers also include but not limited to genetic background, treatment types, glycemic control quality, and disease duration.

Reactive oxygen species (ROS) has been placing a great threat to various tissues/cells, causing pathologies including inflammation, metabolic dysfunction, age-related degeneration, and diabetes. β Cells may be at higher risk of oxidative damage from ROS, as a consequence of excessive levels of mitochondrial ROS generation and reduced nicotinamide adenine dinucleotide phosphate (NADPH) oxidase activity, failure of antioxidant defense, and endoplasmic reticulum (ER) stress (38). Chronic hyperglycemia and hyperlipidemia are characteristic of patients with T2D. Several studies have indicated that mammalian (human and murine) pancreatic islets cultured at high glucose concentration manifested miR-708 upregulation, treated with ER stress reliever, which was known to improve ER folding capacity; however, this upregulation is reversed (30). High levels of free fatty acids (FFAs) can be another trigger for beta-cell oxidative stress. Related miRNA such as miR-34a and miR-146a showed an increasing pattern after palmitate treatment on islet cells, in parallel with increased beta-cell apoptotic behaviors, whose inhibition rescued the viability of beta-cells but not their insulin secretory functions (31, 32). Other miRNA with negative regulation on β-cell survival include miR-182-5p, miR-33, and miR-370, whose effect could be corrected by thrombospondin 1 (THBS−1) and Glucagon Like Peptide-1 (GLP-1), respectively (33, 34).

#### 3.1.2 miRNA and Peripheral Recipient Cells

Insulin resistance is the inability of the target tissues to orchestrate well-coordinated glucose-lowering processes, including the suppression of gluconeogenesis, lipolysis, net glycogen synthesis, and cellular glucose uptake in response to physiological blood insulin levels (70). The glucose-insulin balance is maintained through the liver, skeletal muscle, and white adipose tissue, with the liver exerting major effects as the metabolic center of an organism. Hence, once the hepatic insulin signaling cascade faces impairment resulting in hepatic insulin resistance, other metabolic symptoms occur, incurring hyperglycemia, inflammation, and *de novo* lipogenesis, and further, hepatic steatosis and nonalcoholic fatty liver disease (NAFLD) (67). Thus, it is beyond reasonable that tools for early diagnosis of hepatic insulin resistance is beneficial. miRNAs has been investigated for their potential as biomarkers for hepatic insulin resistance, and to qualify as one, whose circulating levels must correlate to corresponding hepatic states and must be crucial in the signaling cascade. miR-802, an intensively studied miRNA in the oncological field, has been demonstrated to be associated with oxidative stress and hepatic insulin resistance. In high-fat diet (HFD) mice, studies have shown that, along with increased expression of miR-802, ROS generation was significantly greater, and the expression of gluconeogenesis-related genes was significantly downregulated (35, 36), and its circulating levels were dramatically elevated in T2D patients, qualifying it as a biomarker. Other miRNAs with changed expression patterns include miR-499-5p, which affects insulin signaling cascade and glycogen synthesis by suppressing phosphatase and tensin homolog (PTEN), alongside the improvement of Akt/GSK activation, and is found to be reduced in prediabetic patients (19, 20), and miR-122-5p, which affects the hepatic gluconeogenesis process, whose circulating levels were significantly higher in cohorts with insulin resistance, T2D, or MetS (37).

Aside from liver cells, another important target of insulin is skeletal muscle, whose glucose uptake also substantially contributes to glucose and metabolic homeostasis. In an investigation on the antioxidant effects of berberine (BBR), an isoquinoline alkaloid, it was found that BBR could attenuate oxidative stress of diabetic mice partly through inhibiting miR-106b/SIRT1 pathway, an miRNA associated with skeletal muscle insulin resistance (38, 39). Interleukin (IL)-13 has been revealed having an autocrine role in the glucose metabolism of skeletal muscle according to Jiang et al., and its exposure increases skeletal muscle glucose uptake, oxidation, and glycogen synthesis *via* an Akt-dependent mechanism. In T2D patients, such bioactivities are found to be suppressed by the increased expression of miRNA let-7a and let-7d, which repress IL-13 genes on translational level (40). To put it simpler, insulin functions by combining to insulin receptor substrate 1 (IRS-1), subsequently triggering a signaling cascade consisting of phosphorylation of protein kinase B (PKB/AKT), translocation of glucose transporter-4 (Glut4) from the cytosol to the membrane, and glucose uptake. The overexpression of miR-29 was demonstrated to be capable of disrupting the glucose metabolism of skeletal muscle by inhibiting insulin signaling, expression of insulin receptor substrate 1 (IRS-1), and phosphoinositide 3-kinase (41).

Since obesity are getting more and more attention as a risk factor of T2D, the role white adipose tissues play in diabetes is receiving piling investigational highlights. Researchers have found that certain exosomes extracted from obese mice were able to induce glucose intolerance in lean mice transfected with certain exosomes, which was demonstrated to be viable through the miRNA content, namely, miR-192, miR-122, miR-27a-3p, and miR-27b-3p, the expression of all of which was increased in obese mice. Data further showed that the mechanism of induced diabetes in white adipose tissue was navigated by the targeting of peroxisome proliferator-activated receptor α (PPARα) (42, 71).

### 3.2 lncRNA

lncRNAs are a group of transcripts, with lengths extending 200 nucleotides, that also do not translate into proteins. Divided by functions, lncRNAs have three subtypes: the non-functional ones, which serve no purpose other than being by-products of transcriptions; the second type are those whose own transcription is of a self-sufficient manner; and the third type consists of those that are able to act in *cis* and/or *trans* orientations (72). lncRNAs interact intensively with miRNAs, acting as the molecular sponges or decoys of miRNAs to regulate their cytoplasmic level by binding specific miRNAs and actively sequestrate them from their target mRNAs. In turn, lncRNAs can be destabilized by miRNAs’ direct targeting and posed as competitors on shared mRNA targets (67). With such intertwined involvement in gene expression regulation, lncRNAs have been presenting some potential in T2D development and management.

H19 is among the earliest-discovered lncRNAs, whose biofunctions have been relatively thoroughly investigated. A recent study conducted by Sanchez-Parra et al. found that H19 may act upstream of miRNA let-7 and the activation of Akt, whose silencing decreased β-cell expansion in newborns and re-expression promoted proliferation of β cells in adults (43). Moreover, the circulating levels of H19 have been demonstrated to be significantly increased in T2D patient cohorts, according to Fawzy et al., which indicates its potential as a biomarker in insulin resistance (44).

MEG3 is another lncRNA known to researchers for a long time as well, mostly indicated in cancer suppression. Its association has been noted with pancreatic cell apoptosis, insulin synthesis, and secretion, whose expression may function as a new regulator of maintaining beta cells identity (46). However, its overexpression, suggested by Zhu et al., may be a promoter for hepatic insulin resistance *via* increasing FOXO1 expression by acting as a sponge for miR-214 (47). It has also been reported that the competitive binding of MEG3 to miR-185-5p, acting as a competing endogenous RNA (ceRNA), promoted the expression of early growth response 2 (EGR2), which was reported to inhibit IRS. Clinical evidence also supported this hypothesis: its overexpression in T2D patients was significant (45).

Like its counterparts, lncRNAs are associated with oxidative stress-induced insulin resistance as well, according to studies. lncRNA MALAT1, which has been shown to take part in regulation of cell proliferation and motility, has been found to partake in suppressing insulin signaling by inhibiting the phosphorylation of IRS and Akt, *via* the upregulation of the c-Jun N-terminal kinase (Jnk), a stress-sensitive kinase (48). This negative regulatory effect has been corroborated by its increased expression in GDM patients (49).

### 3.3 circRNAs

Circular RNAs (circRNAs) are a group of non-linear, naturally occurring ncRNAs with covalently closed circular structures. They are usually generated from precursor mRNA by a non-canonical event called backsplicing, displaying exceptional stability and evolutionary conservation, and tissue or development stage-specific expression patterns (73). Compared to miRNAs and lncRNAs, where many research results have been obtained, the biological functions discovered for circRNAs are relatively scarce, whereas a few associations have been made between them and glucose metabolism, insulin resistance, and T2D.

Certain circRNAs are capable of fulfilling various intracellular functions mainly by acting as sponges of miRNAs or RNA-binding proteins (RBPs). The best understood endogenous circRNA to this date has been CDR1as (also termed as ciRS-7). By potently binding to miR-7, it relieves the inhibitory effect of miR-7 on β-cell function, subsequently promoting islet β-cells proliferation and insulin secretion (15). Such phenomenon has also been observed *in vivo*, where the expression of CDR1as was reduced in diabetic mice (16). A similar effect has been witnessed in another circRNA, circRNA-HIPK3, whose regulation on islet cell function was mediated by sequestering miR-124-3p and miR-338-3p (16). Clinically, such effect could be potentially employed as a marker for therapeutic outcome evaluation. Reverse transcription polymerase chain reaction (RT−PCR) and quantitative polymerase chain reaction (qPCR) analyses were adopted for the screening of circRNAs related to T2D, and several circRNAs were detected to show statistical significance between T2D patients and healthy controls. Among them, there are hsa_circ_0054633 and circANKRD36, with the former being a potential diagnostic biomarker of prediabetes and T2D in peripheral blood cells and the latter displaying a potential in discerning the T2D-inflicted within cohorts with chronic inflammation (17, 18).

## 4 Discussion

Early detection of diabetes mellitus is very useful for preventing onset and progression. Several biomarkers of diabetes mellitus have been reported, such as GDF-15, YKL-40, 2-aminoadipic acid, serum adipocyte fatty acid–binding protein, and urinary 8-oxo-7,8-dihydro-2′-deoxyguanosine (74–78). While many current biomarkers are based on proteins, ncRNAs have been recognized as a new sensitive, noninvasive biomarker for diagnosis, prognosis, and prediction to therapeutic responses in the recent years due to their high stability in body fluids (urine, plasma, exosomes, etc.) and the development of new detection techniques (79).

miRNAs are very stable and resistant to ribonucleases, freezing/thawing cycles, and other severe experimental conditions (80). Therefore, serum or plasma samples can be stored at −20°C or −80°C for several months without causing significant degradation of miRNAs, which serves as valid evidence to support the use of miRNAs as ideal biomarkers (81). lncRNAs are less stable than miRNAs, but compared to protein-coding mRNAs and miRNAs that are frequently expressed in multiple tissues, they show higher tissue specificity (82) and tend to show remarkable level of overexpression in diabetes. circRNAs are presumably more stable than most linear RNAs because they form a unique, circular, covalently closed continuous loop that is resistant to exonuclease-mediated degradation as they have no 5′ or 3′ ends. Recent evidence indicates that circRNAs usually regulate the transcription of miRNA-target genes by acting as miRNA sponges.

The extensive role of ncRNAs in physiological processes and deregulation in human diseases also makes them very attractive targets for new therapies. Several strategies that either silence overexpressed ncRNAs or reactivate downregulated ncRNAs are currently being investigated. Some of the ncRNAs have even shown encouraging therapeutic effects in animal models. For instance, MEG3 knockdown in STZ-induced diabetic mice resulted in increased levels of acellular capillary formation, microvascular leakage, and inflammatory proteins (83). MIAT knockdown in STZ-induced diabetic rats did not affect body weight or blood glucose levels but corresponded with improvements in visual function and partial reversal of a-wave, b-wave, and oscillatory potentials. MIAT downregulation also decreased the number of apoptotic retinal cells and attenuated retinal vessel impairment and retinal vascular leakage (84). In short, the therapeutic potential of ncRNA has been discovered, but the current findings are only the tip of the iceberg, and further research is still needed.

However, the pivotal issue is how to apply the molecular markers determined in the laboratory to the clinical environment. Currently, various limitations impede this research field and delay the clinical application of ncRNAs. On the grounds that ncRNAs are a quite novel area of research, most of the information obtained from the previous studies is only descriptive and correlational, and it is not possible to precisely infer the cause and effect. In particular, the deficiency of thorough understanding regarding the explicit origin, synthesis, modification, and regulatory pathways, and the interactions, cross-talk and coregulation of ncRNAs may hinder the clinical utilization of these ncRNAs. Hence, more edge leading and precise approaches in ncRNAs expression quantification and functional analysis are required.

According to association studies, prevalent assertion arises that ncRNAs can be used as biomarkers for diabetes. Nonetheless, as mentioned above, correlation does not imply causation; thus, pilot results still require large-scale clinical trials. Furthermore, it is strongly advocated to perform *in vitro*/*in vivo* verification of ncRNAs with candidate predictive functions and therapeutic targets since what occurs under physiological and pathophysiological conditions may not necessarily be consistent with the results from pure calculation and analysis. Therefore, the current data should be regarded as exploratory data and illustrated discreetly before further experiments and comprehensive clinical verification can prove the biological effects of ncRNAs.

The tissue specificity of ncRNAs and their enhanced stability in body fluid also enable them to be a promising future antidiabetic therapy. However, ncRNA therapy that can be authentically applied in the clinics still faces considerable obstacles, including the development of reliable delivery systems, dosage regimens, and technologies to improve off-target effects. On top of these, just like any other therapies, it is expected that ncRNAs as drugs may cause adverse side effects or induce drug resistance.

## 5 Conclusion

ncRNAs have a role in regulation of cellular functions including development, proliferation, differentiation, and apoptosis. ncRNAs anomalies would be indicative as molecular signatures under disease states, which can be applied to arrive at different diagnoses and explore treatments. Considering the rawness of this research field, many ncRNAs functions are waiting to be discovered. In conclusion, studies combining ncRNAs and clinical, genetic, epigenetic, and classical markers could help direct the medical decisions on diabetic patients and show broad prospect in precision medicine.

## Author Contributions

Conceptualization: ZL and PW. Writing—original draft preparation: TC, JL, MW, and YZ. Writing—review and editing: ZL and PW. Supervision: ZL and PW. Funding acquisition: PW. All authors contributed to the article and approved the submitted version.

## Funding

This work was funded by the National Natural Science Foundation of China (Grant No. 31800652), Young Scientist Program by Beijing University of Chinese Medicine (Grant No. BUCM-2019-QNKXJ-C014), and the “Double First Class” Construction Funds of Discipline of Integrated Traditional Chinese and Western Medicine of Beijing University of Chinese Medicine.

## Conflict of Interest

The authors declare that the research was conducted in the absence of any commercial or financial relationships that could be construed as a potential conflict of interest.

## Publisher’s Note

All claims expressed in this article are solely those of the authors and do not necessarily represent those of their affiliated organizations, or those of the publisher, the editors and the reviewers. Any product that may be evaluated in this article, or claim that may be made by its manufacturer, is not guaranteed or endorsed by the publisher.
